# Day-to-Night Street View Image Generation for 24-Hour Urban Scene Auditing Using Generative AI

**DOI:** 10.3390/jimaging10050112

**Published:** 2024-05-07

**Authors:** Zhiyi Liu, Tingting Li, Tianyi Ren, Da Chen, Wenjing Li, Waishan Qiu

**Affiliations:** 1 School of Architecture and Urban Planning, Beijing University of Civil Engineering and Architecture, Beijing 100044, China; 202006010225@bucea.edu.cn; 2 School of Architecture, South Minzu University, Chengdu 610225, China; 202031707075@stu.swun.edu.cn; 3 Department of Product Research and Development, Smart Gwei Tech, Shanghai 200940, China; ymir0203@gmail.com; 4 Department of Computer Science, University of Bath, Bath BA2 7AY, UK; da.chen@bath.edu; 5 Center for Spatial Information Science, The University of Tokyo, Kashiwa-shi 277-0882, Chiba-ken, Japan; liwenjing@csis.u-tokyo.ac.jp; 6 Department of Urban Planning and Design, The University of Hong Kong, Pokfulam Road, Hong Kong SAR, China

**Keywords:** street view imagery, night scene, day-to-night, generative AI, nighttime perception

## Abstract

A smarter city should be a safer city. Nighttime safety in metropolitan areas has long been a global concern, particularly for large cities with diverse demographics and intricate urban forms, whose citizens are often threatened by higher street-level crime rates. However, due to the lack of night-time urban appearance data, prior studies based on street view imagery (SVI) rarely addressed the perceived night-time safety issue, which can generate important implications for crime prevention. This study hypothesizes that night-time SVI can be effectively generated from widely existing daytime SVIs using generative AI (GenAI). To test the hypothesis, this study first collects pairwise day-and-night SVIs across four cities diverged in urban landscapes to construct a comprehensive day-and-night SVI dataset. It then trains and validates a day-to-night (D2N) model with fine-tuned brightness adjustment, effectively transforming daytime SVIs to nighttime ones for distinct urban forms tailored for urban scene perception studies. Our findings indicate that: (1) the performance of D2N transformation varies significantly by urban-scape variations related to urban density; (2) the proportion of building and sky views are important determinants of transformation accuracy; (3) within prevailed models, CycleGAN maintains the consistency of D2N scene conversion, but requires abundant data. Pix2Pix achieves considerable accuracy when pairwise day–and–night-night SVIs are available and are sensitive to data quality. StableDiffusion yields high-quality images with expensive training costs. Therefore, CycleGAN is most effective in balancing the accuracy, data requirement, and cost. This study contributes to urban scene studies by constructing a first-of-its-kind D2N dataset consisting of pairwise day-and-night SVIs across various urban forms. The D2N generator will provide a cornerstone for future urban studies that heavily utilize SVIs to audit urban environments.

## 1. Introduction

### 1.1. Public Space and Safety Perception

Urban public space has significant impacts on the urban ecological environment [1], residents’ physical and mental health [2], urban vitality [3], public life [4], personal identification [5], and city security [6]. Notably, the urban visual appearance, as emphasized by Lynch (1984), plays a crucial role in human perception. For example, the design and maintenance of public spaces hold particular importance to the perceptions of safety, traditionally assessed through various methods, including site observation, questionnaires, surveys, and cognitive mapping [7]. The data collection process involves field surveys, visual auditing (Rossetti et al. 2019), and land use data collection via Geographical Information Systems (GIS) [8].

**Figure A1:**
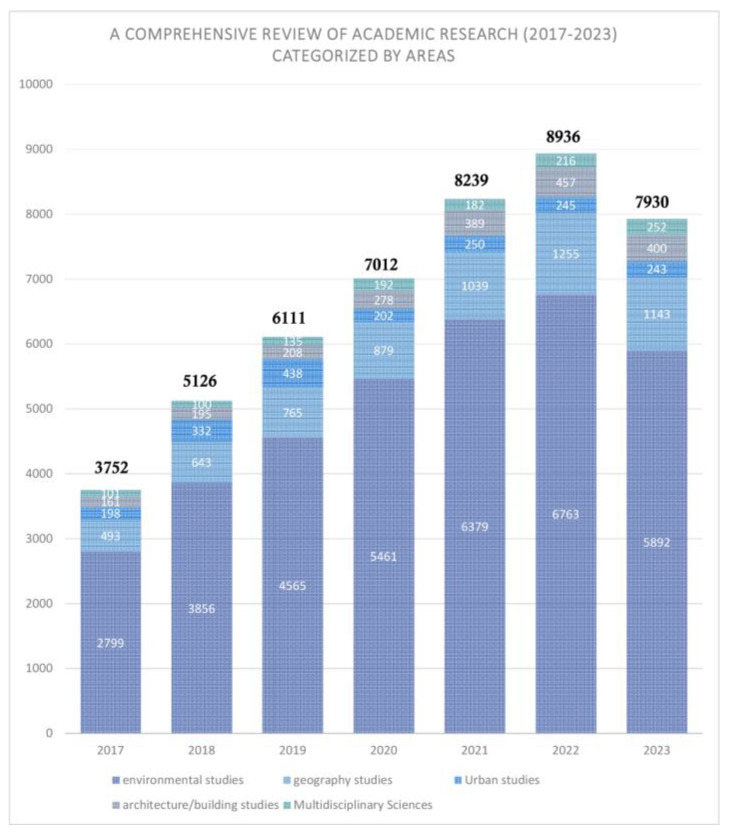
Fast-growing urban studies using SVI.

There has been a notable increase in urban studies addressing urban environmental quality, covering crucial topics including place attachment, urban heat islands, ecosystem services, road traffic, and house prices (Figure A1). Among them, a significant subset (280+ papers) has extensively utilized Street View Imagery (SVI) data [9] and artificial intelligence (AI) models, including machine learning (ML), deep learning (DL), and computer vision (CV) for urban-scale visual auditing [10,11,12]. Emerging studies [13,14,15,16] have indicated that street scene qualities significantly affect human behaviors, including running [17], walking [18], mental health [19,20,21], leisure activities [22], job and housing decisions [23,24], crime [25], and carbon emissions [26].

However, most (~95%) of SVI-based studies exclusively rely on daytime SVIs. Urban night scenes are inadequately explored due to the scarcity of night-time images. This study draws particular attention to the biased urban image database issue. Prior studies failed to describe night-time street environments due to how and when SVIs are collected—SVIs are captured by a car mounted with panorama cameras running through streets during the daytime [27]. That said, unlike the prevalence of the daytime counterpart, no urban-scale night-time SVI dataset exists. This limitation also leads to a deficiency in understanding the variation in day-night perception. For example, a street with high greenery and plantation coverage during the daytime might look pleasant while being scary at night. Commercial streets might look plain and boring during the daytime but prosperous at night. Hence, the absence of night-time images has significantly restricted the comprehensive understanding of urban environments.

Despite the deficits of nighttime SVI, transforming daytime images to night-time ones is not novel in CV studies [28]. For example, CycleGAN [29] has allowed for the production of corresponding nighttime images on a large scale for driverless car studies. Inspired by advancements in CV studies, this study sets out to fill the research gaps.

### 1.2. Knowledge Gaps

First, no urban-scale image dataset exists to provide consistent (i.e., camera settings) and pairwise day–and–night SVIs tailored for night-time street view generation from daytime counterparts for urban studies. For example, CV researchers usually collect pairwise images from non-professional photographs [30,31,32] or render pictures with inconsistent illumination conditions [33,34]. Given the prevalence of urban environment studies using public daytime SVIs, collecting consistent and pairwise day-and-night SVIs is crucial to ensure the generalization ability of day-to-night (D2N) SVI generation.

Second, despite the prevailed D2N conversions in CV studies, little reference exists regarding what models (e.g., StableDiffusion, CycleGAN, Pix2Pix) are more effective for completing the D2N transformation for urban scenes [28,35]. We hypothesize that the capability of pre-trained models in predicting night-time views can be characterized by streetscape variations according to urban form differences. For instance, a transformer model may effectively predict night scenes for low-density urban landscapes (e.g., suburban) while struggles for densely developed central business districts (CBDs). That said, how different GenAI frameworks might perform differently across divergent urban forms is largely unknown, which can provide important references for future studies.

Third, how to consistently evaluate the accuracy of image prediction useful for urban scene studies is unclear, especially regarding how to quantify the divergence between fake and ground-truth nighttime SVIs according to human perceptions. We argue that human perception is more reliable than R2, MSRE, or other common ML error measures, while human validation is important.

### 1.3. Research Design and Contributions

To fill the gaps, this study collected pairwise day–and–night SVIs across various urban forms in four cities to train D2N models. Three popular models (i.e., CycleGAN, Pix2Pix, and StableDiffusion) were tested, and CycleGAN was most effective for the D2N task, a method commonly employed in the automobile industry [28,35]. Notably, streetscape features were extracted with semantic segmentation [36,37] during model establishment for fine-tuning to overcome the bright spot issues. The accuracy of D2N transformation was also evaluated based on human eye preference rating [38] using online surveys to ensure the generalization ability of the night-time scenes generated would be useful for urban scene auditing and human perception measures for future studies. This study established a scientific foundation for policymakers and urban designers to generate night-time scenes for high-throughput urban auditing and management.

## 2. Literature Review

### 2.1. Urban Public Space and Human Perceptions

Urban public space refers to open places accessible to the entire public, mainly used for citizens’ daily lives and social activities, including outdoor spaces such as urban squares, streets, and parks [39]. Public space significantly influences the quality of life [40]. For example, Jane Jacobs emphasizes that quality physical environments, such as well-designed sidewalks, public spaces, and neighborhood stores, can prevent crimes by providing more “eyes on the street” [6]. 

The surrounding environment can affect Night-time perceptions, e.g., the sense of night safety. A few studies evaluated potentially threatening situations, including environmental variables and unsafe environment information [41] and found factors such as darkness, isolation, and desertion can increase feelings of insecurity [42,43,44]. One study illustrated the perceptions of travelers in Hong Kong of the diverged day/night time street view [45]. Another one explored the relationship between street-related elements and fear of crime for females [46]. However, beyond these efforts, very limited studies have focused on perception variations attributed to environmental differences between day and night at the urban scale.

### 2.2. SVI Data for Urban Scene Auditing

Observation, activity notation, questionnaire survey, cognitive mapping, and GPS tracking are commonly used in environmental behavior studies [7]. Some studies collect user preferences by asking interviewees to select preferred pictures and explain their subjective perceptions of public spaces [36,47]. Along this line, the introduction of SVI data dismantled limitations on the accessibility of urban image data sources [48,49], being able to represent characteristics of the built environment at a large scale [9]. SVIs have received considerable attention in urban planning and design [50]. Typically, they can be acquired from a car mounted with cameras on its roof and lidar sensors [51], making them suitable for eye-level urban perception measures [52,53]. It has been used in measuring urban vibrancy, comfort, and attitude towards greenery and safety [13,14,16,20].

The advancements in AI interpretation also enable researchers to decode the subtle correlations between environments and human perception [15,54,55]. CV feature extractors (e.g., GIST, DeCAF ImageNet) have been widely employed to predict perceptual scores from images [56,57]. Most recently, sophisticated methods such as convolutional neural networks (CNNs) also saw increased applications [10,58,59].

### 2.3. GenAI and Nighttime Image Translation

Notably, most images utilized for training originate from public photographic resources. Some studies took an incentive-based method for large-scale photo collection, albeit at the expense of being labor-intensive [32]. However, almost all urban scene studies neglect to collect night pictures. Only certain studies involve rendering images under varying daylight [34] and night illumination conditions. The image quality of a low-light environment also degrades due to color distortions and noise [60,61,62,63]. Fortunately, technologies featuring LLIE [64] and NIR [65] can enhance the image quality in this environment. With the development of deep learning, low-light image enhancement is based on deep neural networks [63,66,67,68,69,70]. With the recent development of generative models, GenAI is promising to allow for converting daytime SVIs into nighttime, contributing to advancements in 24-h street environment auditing and management.

#### 2.3.1. Generative Adversarial Networks (GANs)

Along this line, GANs [71] have emerged as a focal point of D2N research. Its architecture comprises a generative model and a discriminative model. Throughout the adversarial training process, the generative model assimilates the probability distribution of real data, generating synthetic samples capable of deceiving the discriminative model [72]. Specifically, GAN circumvents the explicit definition of pθ(x), which refers to the probability density function (PDF) of variable x, parameterized by θ in the context of generative adversarial networks (GANs), by training the generator through the binary classification capability of the discriminator. The generator is not constrained to adhere to a specific form of pθ(x) [73]. As the generator is typically a deterministic feed-forward network from Z to X, GAN facilitates a straightforward data sampling process, distinguishing itself from models employing Markov chains [74] (which are often slow in computation and imprecise in sampling). Additionally, GAN enables the parallelization of data generation, a feature not feasible in other autoregressive nature models [73].

#### 2.3.2. StableDiffusion

StableDiffusion [75] entails modeling a particular distribution originating from random noise, achieved through both a forward diffusion process and its corresponding reverse diffusion counterpart. The framework is characterized by its emphasis on efficiency, aiming to minimize inference time and computational workload compared to alternative image-generative methodologies [76]. Firstly, the model excels in generating synthetic images characterized by visual realism, effectively capturing a diverse array of conceptual content. Secondly, the generated images serve as valuable training data, facilitating data augmentation within machine learning applications, thereby contributing to enhanced model generalization and robustness. Thirdly, the synthetic images generated demonstrate efficacy in image classification tasks, with certain conceptual representations accurately discerned by vision transformer models, underscoring the model’s discriminative prowess. Ultimately, using synthetic data engendered enriches data diversity in supervised learning settings. This proactive measure mitigates the dependence on labor-intensive labeling processes, presenting a pragmatic solution to challenges associated with data scarcity [77].

However, StableDiffusion has three major cons. Firstly, there exists a challenge in managing the variability in generation speed, where the model encounters difficulties in reconciling disparate rates of image generation across diverse categories. Secondly, the similarity of coarse-grained characteristics emerges as an issue stemming from the entanglement of features at a global or partial coarse-grained level. This phenomenon contributes to generating images with analogous characteristics, hindering diversity. Lastly, the polysemy of words introduces susceptibility into the model, as it incorporates semantically complementary words to the original prompt. This process generates images featuring entirely novel content unrelated to the original category, compromising the model’s semantic fidelity [78].

#### 2.3.3. Day-and-Night Image Translation

Recent developments have introduced a deep generative model designed to transform images between day and night [28], which is common in the automobile industry for vehicles to locate objects and recognize barriers [35]. CycleGAN [29], an approach tailored for translating an image from a source domain to a target domain without the demand for paired examples, is profound in practice. It contributes to the conversion between day and night images. Some researchers have also extended CycleGAN to Pix2Pix and are trying to achieve better performance [79]. Additionally, semantic segmentation plays a crucial role in comprehending the content of images and identifying target objects, especially in the field of automatic driving [37]. Notably, Pix2Pix and CycleGAN are grounded in the family of GAN models. 

Pix2Pix has three pros. Firstly, it utilizes conditional GANs that incorporate a structured loss, penalizing the joint configuration of the output, thereby enhancing the realism of generated outputs. Secondly, using a U-Net-based architecture for the generator facilitates skip connections, directly transferring low-level information between input and output. Thirdly, the PatchGAN discriminator in Pix2Pix focuses on high-frequency structure, resulting in sharper images while relying on an L1 term for low-frequency correctness. However, discernible disadvantages include the potential for blurry results in image generation tasks when employing L1 or L2 loss functions and the limitation of diversity in generated images due to the dropout noise strategy. 

CycleGAN comes with two distinct advantages and one limitation. Firstly, it can learn mappings between two domains without necessitating paired training data, offering increased flexibility in image translation tasks. Secondly, the incorporation of cycle consistency loss aids in preserving the content of the input image during translation, thereby enhancing the overall quality of generated images. Nevertheless, CycleGAN may encounter mode collapse, where the generator fails to capture the full diversity of the target distribution, resulting in limited variability in generated images. Additionally, the absence of paired data in CycleGAN training can lead to training instability and challenges in achieving desired translation outcomes [79].

## 3. Data and Method

### 3.1. Research Design and Study Area

#### 3.1.1. Conceptual Framework

**Figure 1:**
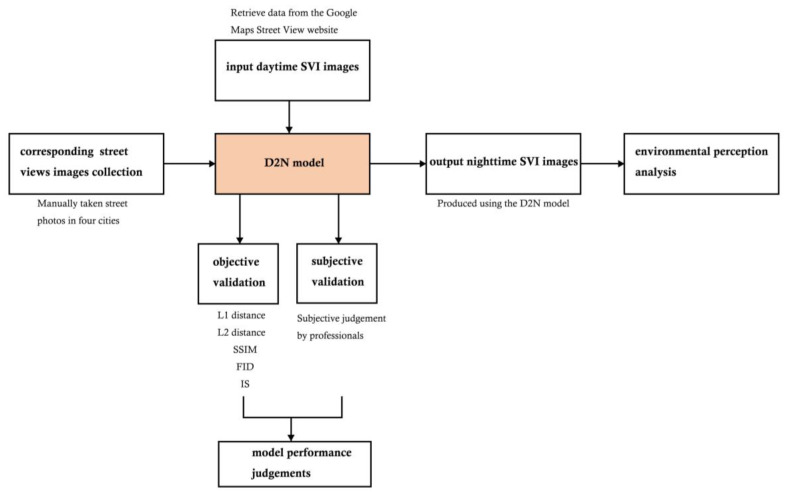
Conceptual framework.

The D2N framework (Figure 1) generates nighttime images from their daytime counterparts. Initially, pairwise day–night SVIs are collected. Subsequently, the model undergoes training utilizing the generative model, followed by a validation process to assess its performance. Then, by inputting an additional daytime SVI without the inclusion of pairwise nighttime images, the D2N model can be employed to obtain nighttime images for the analysis of environmental perception.

The validation process contained two steps. Firstly, common metrics, including L1 distance, L2 distance, and SSIM, were employed to assess the performance of the D2N model by quantifying disparities between real nighttime images and their transformed counterparts [80,81]. Second, three urban designers adept at utilizing SVIs for environmental perception assessment were enlisted. They conducted a comparative analysis of both real and transformed nighttime images, evaluating the transformation performance for their quality.

**Figure 2:**
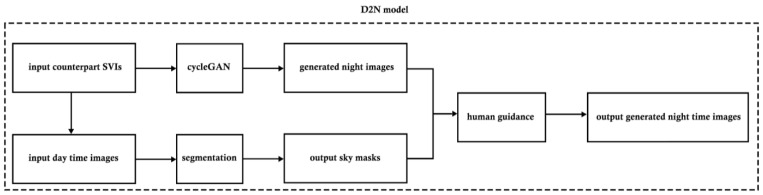
D2N model pipeline.

Figure 2 illustrates the three key steps in the D2N model. Initially, the CycleGAN was employed to generate the basic night images, and the issue of random bright patches in the sky pixels observed in the generated outcomes was addressed by human guidance. Subsequently, segmentation assumed a pivotal role in isolating sky components from their corresponding daytime images. In the third step, the sky masks were combined with the generated nighttime images to improve the overall image quality under human guidance. Ultimately, the D2N model produced the final output of generated nighttime images.

#### 3.1.2. Training and Testing Area

Pairwise day–night street scene photos were sampled from four Chinese cities to construct the training dataset. Once trained, the best D2N model was applied to predict night scenes in New York City (NYC) based on daytime SVI inputs (downloaded from Google Maps) to investigate the model’s viability. NYC was selected as an illustrative case because it exhibits diverse street styles in various areas [82]. The validation of predictions could furnish evidence regarding human perception when encountering streets of varying high-width ratios and architectural styles.

### 3.2. Data

#### 3.2.1. Training Data

The training data were gathered from Beijing, Shanghai, Wuhan, and Chengdu. These images were primarily selected from residential areas with similar street height-to-width and building plot ratios. We carefully controlled the environmental styles of the input training data, striving to avoid specific areas like CBD and wilderness parks [83]. CBD areas and parks exhibit greater variability due to distinct city developments and definitions, lacking universality for our D2N model. In other words, the images excluded skyline buildings and abundant tree coverage, intending to concentrate on environments where people commonly reside. 

**Figure 3:**
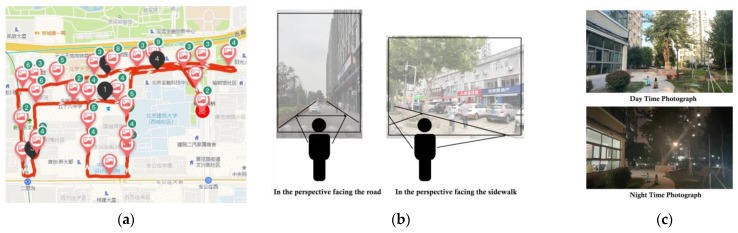
SVI sampling process. (**a**) sampling area in Beijing (The map was captured in Chinese); (**b**) consistent sampling perspective; (**c**) pairwise day/night SVI.

Figure 3a displays the pairwise day–and–night SVI samples gathered in Beijing (106 pairs) within the urban core zone. Figure 3b illustrates how SVIs were collected from the human eye level. The perspective was further categorized into road-facing and sidewalk-facing views. Figure 3c showcases a pairwise day/night scene in a consistent perspective, which ensures optimal performance in training the D2N model. 

All collected pairwise SVIs are used to train and validate progress in CycleGAN. Notably, images were captured during the summer and autumn of 2023. Throughout this timeframe, the presence of greenery remained noteworthy.

#### 3.2.2. D2N Model Efficacy

**Figure 4:**
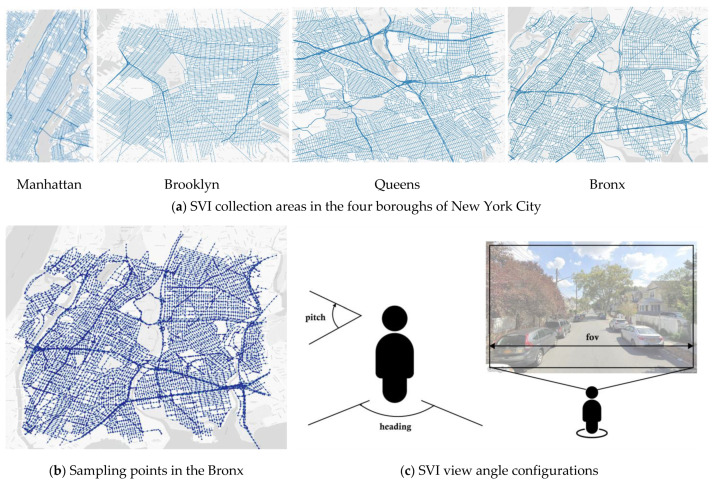
SVI collection (**a**) Four boroughs in New York City; (**b**) Sampling points in the Bronx; (**c**) View angle configurations.

Daytime SVIs were sampled from boroughs of Manhattan, Brooklyn, Queens, and The Bronx in NYC (Figure 4) and occurred at 150 m intervals along the centerlines of public streets utilizing QGIS (Figure 4). The shapefile of the road network was obtained from OpenStreetMap [84]. 42,306 points were sampled, and an 800-point subset was randomly selected to request SVIs with Google Street View API [85].

Identical camera settings and image resolution were set to maintain a consistent viewing angle with three API parameters. The “heading” (view direction) was aligned parallel to the street centerline, the “FOV” (horizontal field of view) was set at 90 degrees, and the “pitch” (the up or down angle of the camera) was maintained at 0 degrees. Additionally, the resolution was standardized at 640 × 400 pixels (Figure 4). For each SVI point, only the view parallel to the tangent of the street centerline was downloaded—a perspective previously employed in urban design studies [86]. 

Notably, even though a substantial proportion of SVIs were oriented parallel to the street centerlines, images captured from the sidewalk perspective could still transform. This capability is attributed to including this specific perspective in our training input images.

### 3.3. Model Architecture

#### 3.3.1. Generative Models

Three distinct GenAI models—CycleGAN, Pix2Pix, and Stable Diffusion—were utilized, and their results will be presented in Section 4.1. Based on performance measures, CycleGAN was selected to execute the translation process. It is not reliant on paired training examples. It has proved versatile and applicable to a broad spectrum of image-to-image translation tasks, especially when acquiring paired data posed challenges or incurred substantial expenses. Consequently, it found practical application in transforming nighttime SVIs from their daytime counterparts.

#### 3.3.2. Semantic Segmentation for Adjustment

Notably, night-time images generated by CycleGAN contained evident sporadic bright patches, primarily concentrated in each image’s sky regions. Semantic segmentation was employed to gain a more nuanced understanding of the image. This technique, as outlined by Zhou et al. [87,88], facilitates the partitioning of the image into semantically meaningful regions, each corresponding to a distinct object or class. By employing this method, we could isolate the sky components within the images, allowing us to focus on the predominant bright patches in the sky for subsequent refinement during human-guided steps.

### 3.4. D2N Model Training

**Figure 5:**
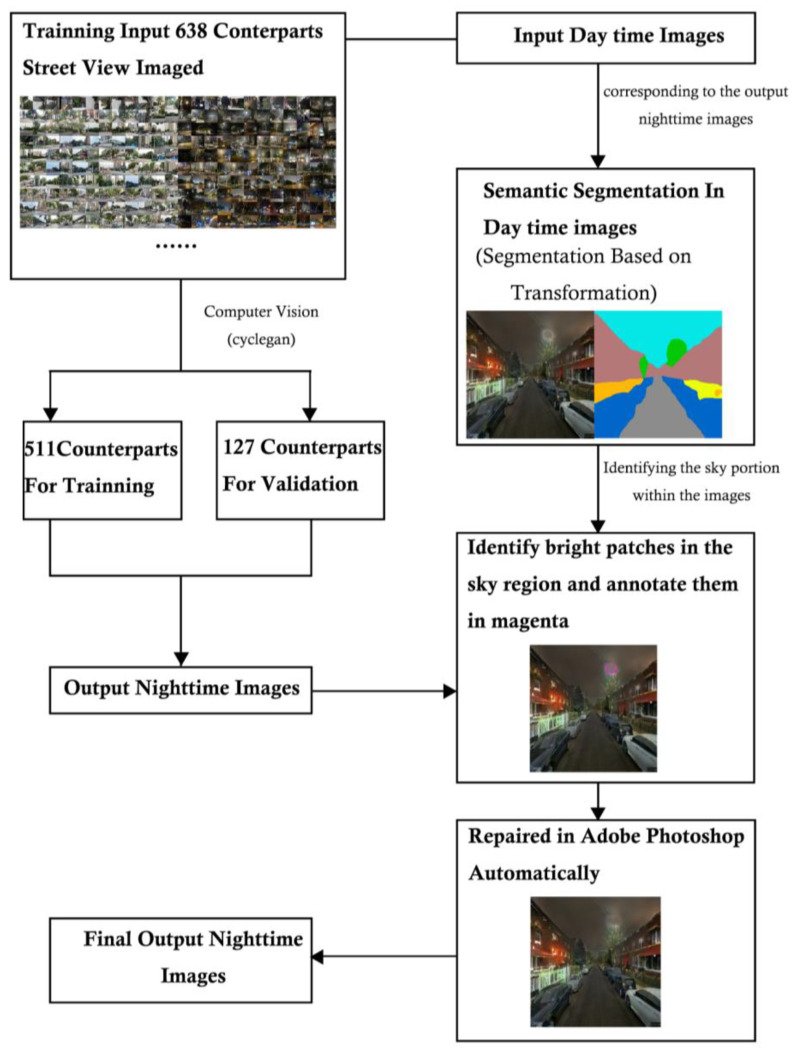
Model training framework.

The model training process was fivefold (Figure 5). First, 638 pairwise day–night SVIs were split into training and validation subsets. Second, CycleGAN [29,79] was employed to train D2N transformation. Third, we adopted semantic segmentation to separate the sky pixels, enabling further automatic identification of bright pixels. Finally, we corrected the masked pixels in Adobe Photoshop using batch processing. We integrated the bright patches into the seamlessly manipulated sky through content recognition.

### 3.5. Model Performance Validation

#### 3.5.1. Objective Judgements in Model Performance

L1/L2 distance and SSIM are metrics to explore the differences between real and fake nighttime images. It quantified cumulative absolute disparities among corresponding elements within two vectors. In the realm of images, when two images are articulated as vectors of pixel values sharing identical dimensions, the computation of L1 distance involves aggregating the absolute distinctions between their corresponding pixels. For GANs, it frequently serves as a metric for evaluating the faithfulness of generated images concerning content. The endeavor to minimize the L1 distance between generated and authentic images aims to enhance the proximity of the generated images to their authentic counterparts on a pixel-by-pixel basis. The L1 distance can be computed by

(1) $$L1\;\mathrm{distance}=\sum_{i=1}^{n}|x_{i\;}-y_{i}|\;$$

- *n*: the dimensions of vectors *x* and *y*, indicating the number of elements they contain;
- $x_{i\;}$ and $y_{i}$ represent the *i*th element of vectors *x* and *y*, respectively;
- L1 represents the L1 distance between *x* and *y*, also known as the Manhattan distance, which is the sum of the absolute differences of corresponding elements in the two vectors.

L2 distance (i.e., Euclidean distance) calculates the square root of the sum of squared differences between corresponding elements of two vectors. The image domain measures the overall dissimilarity in pixel values between two images. In GANs, it is commonly used to evaluate generated images’ overall structure and color distribution. Minimizing the L2 distance between generated and real images helps to ensure that the generated images are globally closer to the real ones. The L1 distance can be computed by

(2) $$L2\;\mathrm{distance}=\sqrt{\sum_{i=1}^{n}{\left(x_{i}-y_{i}\right)}^{2}}$$

- *n*: the dimensions of vectors *x* and *y*, indicating the number of elements they contain;
- $x_{i\;}$ and $y_{i}$ represent the *i*th element of vectors *x* and *y*, respectively;
- L2 distance represents the L2 distance between *x* vectors and *y*, also known as the Euclidean distance, which is the square root of the sum of the squares of the differences of corresponding elements in the two vectors.

Structural similarity index (SSIM) is a method for assessing image quality. It involves the computation of three key components: luminance, contrast, and structure comparison. The luminance comparison function assesses the likeness in average luminance values, while the contrast comparison function evaluates the similarity of contrast values. Simultaneously, the structure comparison function quantifies the structural similarity between signals. Combining these components generates the comprehensive SSIM index, allowing an overall similarity measure. This index is defined by adjusting the relative importance of the three components through parameters, ensuring that properties like symmetry, boundedness, and a unique maximum are satisfied. The SSIM index algorithm can be implemented using MATLAB [89].

Inception score (IS) serves as a pivotal evaluation metric for assessing the quality of generative adversarial network (GAN) models [90]. It emerges as a response to the conspicuous absence of a definitive evaluation metric for GANs, aiming to furnish a standardized yardstick for comparing the efficacy of diverse models in producing authentic-looking images. Notably, IS demonstrates a notable alignment with the human perceptual judgment of image fidelity, underpinned by its design to quantify the salient objects present within generated images. Moreover, IS closely intertwines with the optimization objectives employed during the training phase of generative models, thus manifesting as a robust metric that aptly mirrors human evaluative criteria [90].

On the other hand, Fréchet Inception Distance (FID) represents a pivotal measure for quantifying the dissonance between the statistical distributions of real-world samples and their synthetic counterparts generated by GANs. FID discerns the dissimilarity between these distributions by assessing the Fréchet distance between their respective means and covariances. FID offers a quantifiable measure of dissimilarity, encapsulating the divergence between the distribution of model-generated samples and real-world samples [91].

#### 3.5.2. Human Validation

The objective of our D2N model is to inform night scenes to audit environmental perception. During perception auditing, experimenters typically engage in subjective observation of entire images. Consequently, relying solely on pixel-based measures proves inadequate for evaluating the human eye and perception.

Therefore, subjective Image Quality Assessment (IQA), a method used to evaluate the performance of image processing algorithms, is also deployed. Observers make quality judgments on the assessed images based on predefined evaluation criteria or their own subjective experiences, assigning quality scores according to visual effects. Ultimately, the average subjective score of the image, known as the mean opinion score (MOS), is obtained by weighting the scores given by all evaluators and calculating the mean. A higher MOS score indicates better image quality. By calculating the MOS across all 127 sets of images, we can derive subjective values to assess the model’s performance.

**Figure 6:**
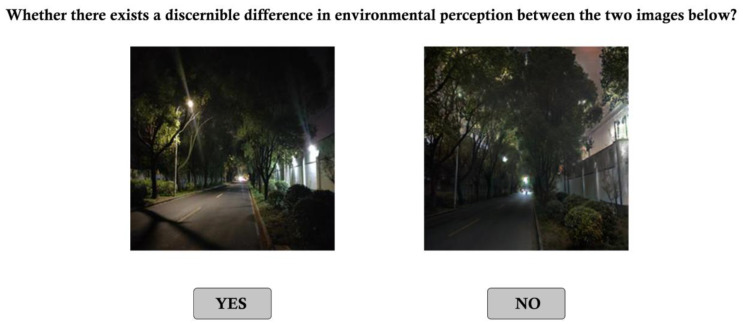
The survey interface.

Three undergraduate student research assistants participated in a 4-h training. Three professionals who previously utilized SVIs to audit environmental perception participated in our study. The three professionals majored in different areas, including urban design, architecture, and technology. The raters were presented with two nighttime images exhibiting subtle differences. One image was an authentic nighttime photograph captured in the real world, while the other was generated by transforming a daytime image using the D2N model. The observers were instructed to simultaneously observe both images and assess whether they perceived varying degrees of environmental perception in the two images. Figure 6 illustrates the survey interface. The response “YES” indicated a discernible difference in perception between the two images, whereas the response “NO” suggested that the two images appeared similar in perception.

They participated in the experience over two days, with sessions arranged in the morning on the first day and in the afternoon on the second. We conducted a comparative analysis of the results from each set of images captured on both days. 

The inter-rater reliability analysis, which measures the agreement rate between observers, was conducted, indicating a score of 56.69% (Table 1). Regarding the agreement across different periods for each rater, the Intra-Rater Reliability (IRR) analysis was conducted, resulting in a 90.3% average intra-rater reliability score, with observed agreement rates of 95.3%, 81.9%, and 93.7% for the three raters, respectively. 

Table 2 presents the Intraclass Correlation Coefficient (ICC) values, i.e., the analysis of within-group correlation coefficients whose values range between 0 and 1. Their confidence intervals and F-test results are also listed, illustrating the consistency and absolute agreement measured by the single-measure ICC (1,1) and average-measure ICC (1,k) methods. Interpretation of ICC is typically as follows: <0.2 indicates poor consistency; between 0.2 and 0.4 indicates fair consistency; within 0.4 to 0.6 indicates moderate consistency; between 0.6 and 0.8 implies strong consistency; and between 0.8 and 1.0 signifies strong consistency. This study’s poor performance of the single-measure ICC (1,1) is reasonable, primarily because our classification is limited to two categories.

### 3.6. Validating D2N with NYC Street Scenes

Applying the D2N model to transform daytime SVIs in NYC into nighttime ones indicates that D2N performance varied significantly across urban forms. Therefore, we aimed to establish a connection between the streetscape features in SVIs and the model’s accuracy: (1) Which urban styles would yield better performance in our training model? (2) What features within the entire images significantly impact the transformation performance? (3) How do these features influence the model’s performance?

### 3.7. Quantifying Impact of Streetscape Elements on D2N Accuracy Using OLS

We hypothesize that the performance of our model can be predicted by regressing the accuracy against various component features present in the input daytime images. The ordinary least squares (OLS) modeling consists of three steps. Initially, we employ semantic segmentation to identify each component in our model. A view index is conventionally construed as the proportion of a feature’s pixels relative to the total pixels within an SVI. An illustrative example is the sky view index, denoting the percentage of sky pixels within an SVI. These view indices inherently encapsulate the significance of visual elements within the pedestrian’s eye-level perspective. Consequently, the quantification of various physical features in SVIs is achieved by applying the general formula (3). We utilized a semantic segmentation model based on the transformer to derive each component from the images [87,88].


(3) $${\mathrm{VI}}_{\mathrm{obj}}=\frac{{\Sigma_{i=1}}^{n}PIXEL_{obj}}{{\Sigma_{i=1}}^{m}PIXEL_{total}}\;,\;\mathrm{obj}\epsilon \left\{tree,building,sky,etc\right\}$$

- *n* represents the total number of pixels in the object of interest;
- *m* represents the total number of pixels in the entire image;
- *PIXEL_obj_* represents the number of pixels in the object of interest, which is the sum of all pixels belonging to the object of interest;
- *PIXEL_total_* represents the total number of pixels in the entire image, i.e., the sum of all pixels;
- *obj ∈ {tree, building, sky, etc.}* represents the categories of the object of interest: trees, buildings, sky, etc.

Second, we constructed a baseline model using the component rate in each SVI and the corresponding objective metric results. The L1 and L2 distance values, approximately 100, were proportionally scaled to a range between 0 and 1 before establishing the OLS model.

Third, we computed the variance inflation factor (VIF) to assess variables for correlation issues (VIF value > 10). Subsequently, less important variables exhibiting multicollinearity (VIF > 10) were removed.

## 4. Results and Findings

This study aims to fill gaps in urban nightscape photo data by converting daytime photos into nighttime photos. This section will detail the results presented in Section 3.3 and present our findings.

### 4.1. Comparison of Three GenAI Models 

Before converting day scene photos into night scene images, we established criteria to assess the generated effects’ quality and the results’ desirability. First was a crucial standard that ensured that all elements in the daytime scene remained intact and identifiable following the conversion to nighttime. This is particularly pertinent for key elements such as streets, which should maintain their original width without distortion. Similarly, other objects, such as trees or houses, should not be significantly changed.

The second criterion is that the light, shadow, and sky generated by the night scene need to match the real night scene. The generated nighttime scene should closely resemble an authentic nighttime environment. We employed objective and subjective evaluation methods to assess compliance with the criteria, as elaborated in Section 4.2 and Section 4.3.

With the judgment criteria established, we initiated the generation of nighttime scenes by experimenting with three prominent models involving image generation and conversation tasks: Pix2Pix, StableDiffusion, and CycleGAN.

**Figure 7:**
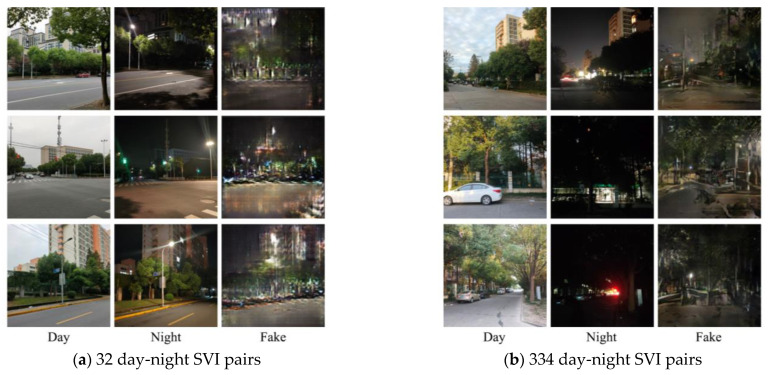
Pix2Pix-generated results are based on different data amounts: (**a**) 32 pairs and (**b**) 334 pairs. Note: Day scene (Day), real night scene (Night), generated night scene by Pix2Pix (Fake).

The generative adversarial network (GAN) is a powerful deep learning model, demonstrating remarkable efficacy in various tasks such as image generation, style transfer, and image conversion. Initially, we opted for Pix2Pix, one of the variants of GAN, anticipating that the final generated results would exhibit qualitative improvement as the dataset expanded. Contrary to expectations, the qualitative leap anticipated with dataset expansion did not materialize (Figure 7).

**Figure 8:**
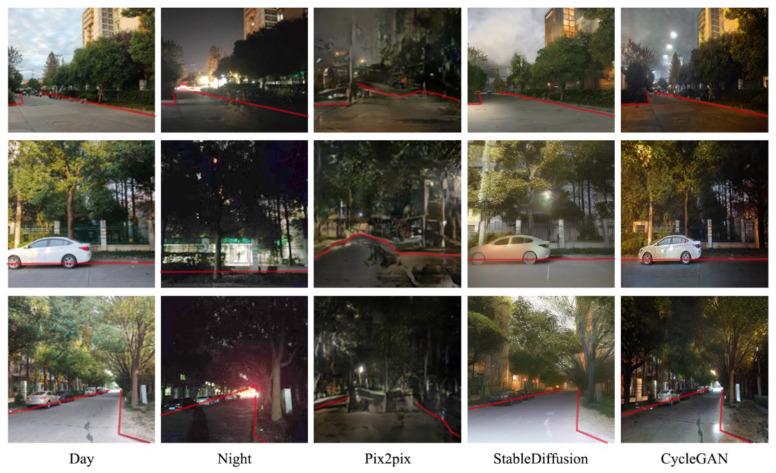
Pix2Pix vs. StableDiffusion vs. CycleGAN (red line: street outline. StableDiffusion and CycleGAN maintain consistency with elements of real scenes, while Pix2Pix distorts features. CycleGAN’s images have lower brightness, resembling real night. Overall, CycleGAN is most effective in retaining more night scene details).

Meanwhile, we explored the StableDiffusion method, which also demonstrated proficiency in preserving the elements of daytime scenes for more realistic night scenes. However, it tended to modify certain details and introduce random lights that did not align with real night scene photos (Figure 8). Consequently, our attention shifted to another GAN variant, CycleGAN.

To our surprise, with the same sample size, CycleGAN significantly outperformed Pix2Pix regarding generation quality. Pix2Pix failed to preserve the distinct streetscape features present in daytime scenes, generating noticeable distortions, and the boundary outlines of houses, roads, and trees were blended. For instance, a white car visible in the daytime scene disappeared in the night view produced by Pix2Pix, while it remained discernible in the night scene by CycleGAN. This underscores CycleGAN’s ability to retain the essential elements of daytime scenes when generating night scenes (Figure 8). Moreover, as the dataset size increased, the final generation effect demonstrated continuous improvement. This also verified the content of the research [79].

In contrast, the night scenes generated by CycleGAN were more in line with the real-world conditions. Thus, we chose to utilize CycleGAN to train the D2N model.

### 4.2. Model Accuracies in Subjective and Objective Assessments

We conducted a comprehensive validation of the model’s accuracy through both objective and subjective evaluations. For objective verification, we employed L1 distance, L2 distance, SSIM, FID, IS, and IS_std. as metrics. Small values for L1 and L2 distances and SSIM values closer to 1 indicated better model performance. However, as shown in Table 3, the SSIM value did not exceed 0.5, which is below the desired threshold in the objective evaluation system. For the metrics, FID and IS, FID was mainly aimed at measuring the feature distance between individual samples. A low FID value means that the generated images were of higher quality because their feature distribution was more similar to the real images. IS was aimed at measuring the overall distribution of the real images. A higher IS score indicates that the images generated by the D2N model were better in terms of quality and diversity. According to the various metrics, the D2N model demonstrated superior performance in facilitating day-to-night transitions compared to alternative models. Additionally, we substantiated the feasibility of the model through subjective evaluation.

We invited three professionals experienced in using SVI for environmental perception auditing to compare pairwise nighttime images—one taken in real life and the other generated from the corresponding day-scene photo using the D2N model. The evaluation focused on discerning different levels of environmental perception within the two sets of images. The MOS from 127 images was finally obtained at 51.18%. The subjective assessment indicated a more favorable perception of the generated photos than the objective evaluation results.

### 4.3. Divergence between Subjective and Objective Evaluations

Human perceptions are intricate, creating potential correlations among diverse perceptual attributes. The subjective evaluation reveals a more positive effect in generating photos than the objective assessment. This is reasonable because environmental perception encompasses various factors, such as the sense of enclosure scores, greenness, etc. The transformation of night scenes has a limited impact on these aspects, resulting in minimal influence on the subjective level. However, because objective evaluation is judged through pixel differences, these factors can influence the final judgment and contribute to variations in subjective and objective assessments. Such divergences between the two measurement systems imply that the underlying mechanism of subjective perception would be quite different from the objective formulas. Unobserved factors cannot be captured by simply summing up or recombining view indices of selected visual elements. The D2N model can compensate for the gaps in night scene photos, particularly in urban areas.

### 4.4. Impact of Streetscape Elements on D2N Transformation

**Figure 9:**
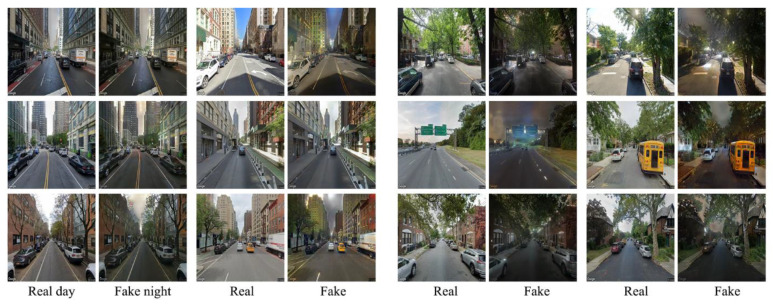
Manhattan vs. Queens (real day view of Manhattan and its corresponding generated night view (left), and the real day view of Queens and its corresponding generated night view (right)). Manhattan’s street scene is characterized by many tall buildings and a small street D/H ratio, resulting in a subpar final generation effect. On the contrary, Queen’s street scene closely resembles the dataset, resulting in a nighttime effect that closely mirrors reality.

For the night scenes generated in the Bronx, Brooklyn, Manhattan, and Queens, the ones in Queens exhibited the most favorable conversion outcome, whereas those of Manhattan were comparatively inferior (Figure 9). In examining the similarities and differences between these two regions, we found certain characteristics in the daytime images—for example, the street’s architecture form or the sky’s proportion—affect the final generation outcome. 

Therefore, semantic segmentation was conducted to quantify the proportion of each streetscape element within the input daytime SVI. An ordinary least squares (OLS) model employed these proportions as independent variables. We utilized the L1 distance, L2 distance, and SSIM as dependent variables, representing the disparity between the generated image and the real night scene. Notably, VIF calculations indicated an absence of collinearity when VIF < 10. We took the most tolerant criterion and removed continuous variables with VIF ≥ 10.

As depicted in Table 4, no element significantly affected L1 distance. Fences and sidewalks impacted the L2 distance, while buildings and the sky contributed to variation in the SSIM. This suggests that the proportion of sky in the input photos, architectural style, and building height are pivotal in influencing the output photo’s quality. For example, a unit increase of sky view corresponds to a notable increase of 0.3496 units in SSIM. This quantitative insight highlights the significant impact of the sky-related characteristics on the perceived structural similarity of the generated night scene photos.

**Figure 10:**
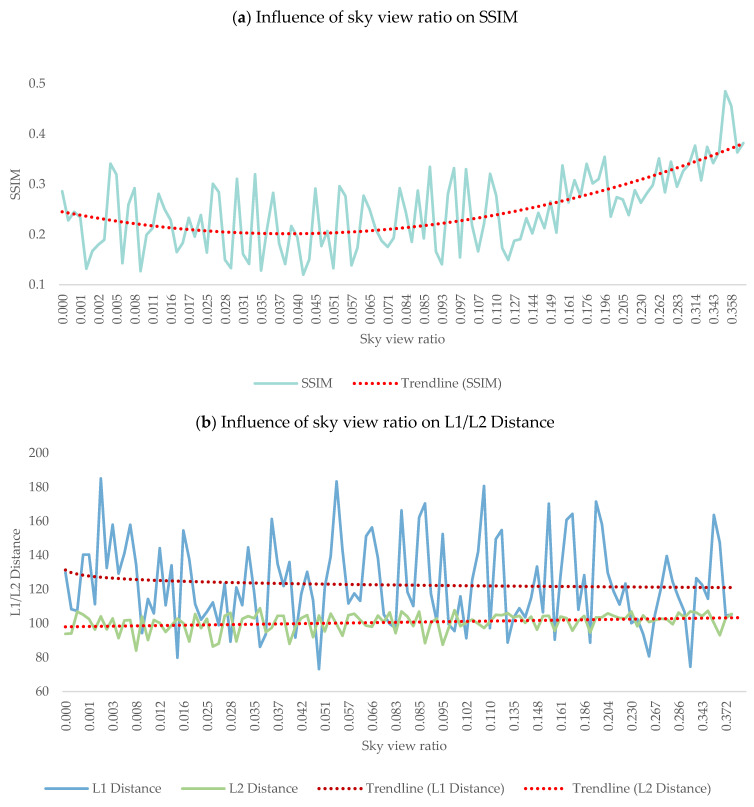

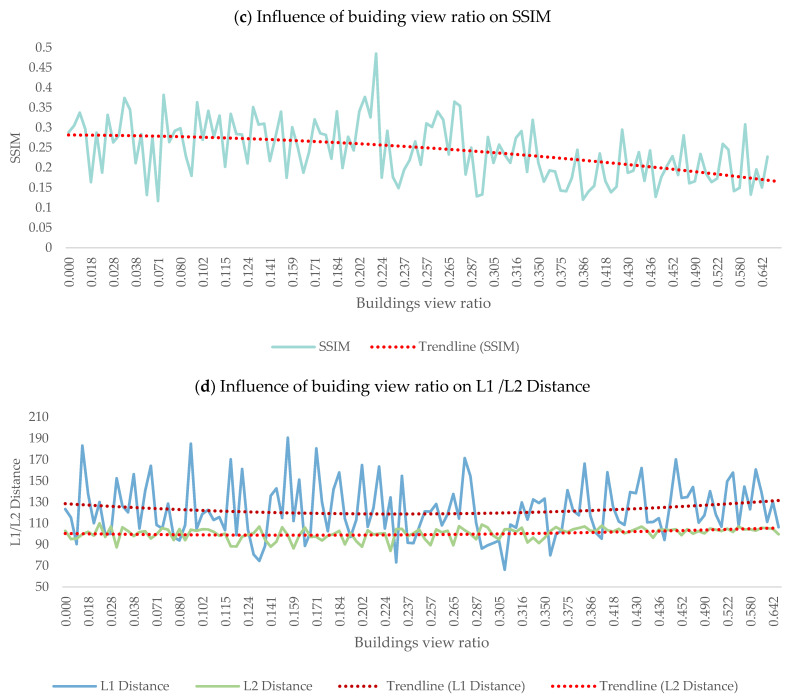
Impact of sky/building view ratio on SSIM and L1/L2 distances.

Figure 10 reports the influence of the sky and buildings on night scene generation. Table 5 and Table 6 present the L1 Distance, L2 Distance, and SSIM values corresponding to three scenarios: the smallest, medium, and largest ratios for the sky and building views, respectively. While the ratio had no obvious effect on the L1 Distance or L2 Distance, the SSIM values indicated an improvement in generation quality with the increasing sky ratio up to a certain threshold. When the building ratio increased, the generation effect became worse.

### 4.5. Improving the Dataset

#### 4.5.1. Using CycleGAN to Generate and Transform Night Scenes

After completing the dataset’s organization and achieving a viable model through training, the transition to generating night scenes from daytime photos was initiated. However, within this process, we encountered and addressed a noteworthy challenge.

The notable challenge arose when we observed a discrepancy in the number of input photos compared to the corresponding output during the generation process. Despite inputting 800 daytime scenes in each region, the generated number of night scenes was less than the expected 800. It became evident that certain photos were skipped in the generation process, leading to incomplete outputs.

In response to this issue, a proactive measure was taken to address the shortfall in output. We sought to supplement the generated night scenes by downloading additional SVIs from Google Maps for the areas requiring augmentation. This approach compensated for the missing outputs and enriched the dataset for further research in the future, fostering a more comprehensive and representative set of nighttime scenes.

This problem, though presenting a challenge, prompted a strategic intervention that resolved the immediate issue and contributed to our dataset’s overall robustness. By downloading more additional SVIs, we not only mitigated the shortfall in output but also expanded the diversity and inclusivity of our dataset, enhancing the efficacy and reliability of our night scene generation process. This adaptive approach reinforces the dynamic nature of our methodology, ensuring its resilience and applicability across the datasets.

In summary, improvements to our night scene generation process involved insufficient generated image data. Through strategic measures such as systematic renaming and increasing the number of input photos, we successfully elevated the precision and reliability of our model, ensuring that the final output aligned harmoniously with the intended input, thereby fortifying the efficacy of our approach to generating realistic night scenes from daytime photographs.

#### 4.5.2. Ways to Make Night Scenes More Realistic

After successfully generating night scene photos, an observation emerged concerning areas characterized by clouds and high daytime sky brightness. In such locations, the final output photos exhibited bright spots significantly incongruent with the intended night scene ambiance. Recognizing the need for additional processing, we implemented a mask code, as mentioned above, to identify and rectify these inconsistencies.

The repair involved setting a masking range and coloring the identified bright spots in a distinct magenta for subsequent corrective measures. Configuring the threshold to “target_th = 125” effectively blocks the brightest spots in the generated night scenes. However, some bright spots persisted in the images despite this threshold setting. 

To address the bright spot issue, we filtered out the problematic portions of the picture and adjusted the threshold to “target_th = 100”, expanding the range of the magenta marking. This subtle adjustment allowed for comprehensive coverage of bright spots while minimizing the impact on other sky segments (that were not problematic). This step corrects the bright spots in final night scenes with precision and minimal disruption to the overall image by fine-tuning the threshold and marking range.

## 5. Discussion

### 5.1. Generating Night Scenes

Our findings indicate that the compatibility between features of the input photo and those within the training dataset is crucial for producing favorable night scenes. The training focused on transforming daytime photos into nighttime equivalents, bridging the substantial gap in night scene data scarcity. This effort contributes to the enrichment of night scene databases and potentially addresses research gaps in understanding urban safety during nighttime for future studies.

### 5.2. Model Accuracy

Judgments about model accuracy are affected by subjective and objective factors. Still, subjective and objective judgment results differ because the intricate and all-encompassing facets of the human sensory process often lead to subjective perceptions encompassing variations rather than consistent coherence. Subjective perceptions sometimes have opposite implications when compared to objective perceptions. Therefore, a single subjective perception has many factors that contribute to the complexity of our understanding of urban scenes. It can represent the subtle sensory processes through which humans interpret urban environments more comprehensively, exhibiting heightened explanatory prowess to many human behaviors.

The influence of the sky and buildings on the generated effect cannot be ignored. The photo’s proportion of the sky and buildings is similar to the street H-W ratio, which is related to preparing the dataset. A notable correlation was identified between the generated images’ quality and the training dataset’s characteristics. It can be observed that the urbanscape characteristics within the training images are critical to the accuracy of the prediction.

### 5.3. Limitations

The data utilized in this study primarily originate from low-density urban areas, covering street intersections, along-street pathways, and roadside trees. Most of these data share the following characteristics: (1) inclusion of partial building structures, (2) high vegetation coverage, and (3) presence of roadways.

Due to the similarity in the data, the night scene generation in low-density urban areas with the mentioned features demonstrates good adaptability. However, distortions may occur in high-density urban areas without buildings and scenes with special structures. These areas exhibit the following characteristics: (1) excessively high or low sky proportion; (2) low vegetation coverage; (3) absence of roadways; (4) low street height-to-width ratio; and (5) presence of special structures such as elevated bridges.

To enhance the realism and diversity of generated night scene photos, refining the dataset during the model training stage by including photos from high-density urban areas, areas without buildings, and scenes with special structures is imperative.

## 6. Conclusions

### 6.1. CycleGAN Demonstrates Best Adaptability for D2N Transformation

Comparing the three models (StableDiffusion, Pix2Pix, and CycleGAN), images generated through Pix2Pix exhibited a non-negligible amount of distortion and blurriness. In contrast, night scenes generated by CycleGAN preserved streetscape elements more completely and clearly. Meanwhile, StableDiffusion yields high-quality images with expensive training costs. The comparison highlights the versatility of CycleGAN, which can perform image transformations between two domains without requiring one-to-one correspondence in training data pairs. Therefore, CycleGAN is more suitable for flexible image transformation tasks like style transfer and seasonal changes without a clear one-to-one mapping. CycleGAN does not require additional cycle consistency constraints, which are built into the model structure as one of the inherent losses, eliminating the need for extra constraints and aiding in maintaining consistency in image transformations.

### 6.2. Urban Density or the Height–to-Width (H-W) Ratio of Streets Are Crucial

The subjective evaluation data surpassed objective assessments, with photos featuring moderate sky visibility and lower H-W ratios standing out in terms of generation effectiveness (Table 4). This reinforces the idea that sky visibility and the H-W ratio of streets are pivotal factors in night scene transformation. This is because we primarily sampled daytime SVIs from low-density building areas. Therefore, the training dataset was characterized by a moderate view ratio of sky and street and higher greenery, indicating lower street height-to-weight ratios [84]. As a result, NYC scenes with moderate levels of sky visibility and lower H-W ratios exhibited better adaptability to the generation of night scenes. That said, one effective way to enhance the accuracy of D2N transformation for future studies is to diversify the training dataset with SVIs from different urban densities. 

## Figures and Tables

**Table 1:** Reliability of human validation.

Category	IRR	Average IRR	ICC Values	
	% Agreement	% Agreement	Single-Measure ICC (1,1)	Avg-Measure ICC (1,k)
The discernible difference in perception	56.69	90.30	0.226	0.467

**Table 2:** Result of the ICC.

	Within-Group Correlation	95% Confidence Interval		F-Test for True Value 0			
		Lower Bound	Upper Bound	Value	df1	df2	*p*-Value
Single Measure ICC (1,1)	0.226	0.117	0.343	1.879	126	254	0.000 ***
Avg-Measure ICC (1,k)	0.467	0.285	0.61	1.879	126	254	0.000 ***

Note: ***, **, * represents significance levels at 1%, 5%, and 10% respectively.

**Table 3:** Metrics to evaluate the models’ efficacy.

	L1		L2		SSIM		IS		FID
	Avg.	S.D.	Avg.	S.D.	Avg.	S.D.	Avg.	S.D.	Avg.
Pix2Pix	129.70	23.88	101.79	5.60	0.22	0.07	1.86	0.08	178.68
CycleGAN	123.03	25.53	100.53	5.39	0.24	0.07	2.54	0.18	115.23
Stable Diffusion	141.74	13.03	104.75	2.08	0.18	0.07	2.41	0.33	156.17
D2N (ours)	122.89	25.73	100.54	5.38	0.24	0.07	2.48	0.31	115.17

Notes: (1) Smaller L1/L2 distances, SSIM being closer to 1, lower FID, or higher IS indicate better D2N transformation performance. (2) IS_std indicates the volatility of the model.

**Table 4:** OLS results between D2N error and streetscape elements.

		OLS Coefficients		
Variables	VIF	L1 Distance	L2 Distance	SSIM
Constant	/	134.7943	0.5100	0.2755
Building	4.42	−17.5975	0.2874	−0.1753 ***
Earth	1.05	−306.7485	−0.6346	0.6700
Fence	1.10	−100.0013	1.8497 ***	−0.4104**
Grass	1.25	−25.5067	−0.8303	0.3663 **
Plant	1.39	−37.3540	0.8065 **	−0.0471
Sidewalk	1.29	66.4387	0.7130 **	0.0156
Sky	3.06	−24.9585	0.6250 ***	0.3496 ***
Tree	4.58	−14.9638	−0.3477 *	−0.0675
Wall	2.84	−11.2872	0.1433	−0.0319

Note: ***, **, * denote significance level of 1%, 5% and 10%, respectively.

**Table 5:** Impact of sky view ratio on night scene generation.

Day/Real Night/Generated Night	Sky View	L1	L2	SSIM
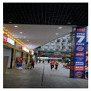 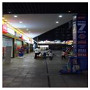 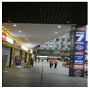	0	130.1384	110.0487	0.1879
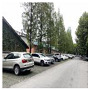 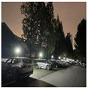 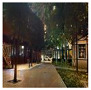	0.166	128.6256	104.0358	0.2639
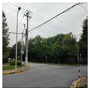 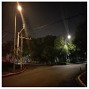 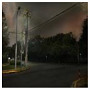	0.3725	147.409	93.0938	0.4559

**Table 6:** Impact of building view ratio on night scene generation.

Day/Real Night/Generated Night	Building View	L1	L2	SSIM
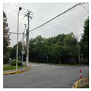 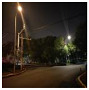 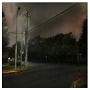	0	147.409	93.0938	0.4559
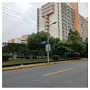 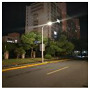 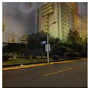	0.2639	108.0863	101.4729	0.34041
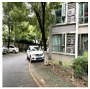 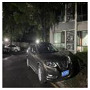 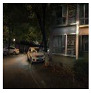	0.6231	139.394	105.7735	0.1330

## Data Availability

Data, models, or codes supporting this study’s findings are available from the corresponding author upon reasonable request.
